# Risdiplam treatment following onasemnogene abeparvovec in individuals with spinal muscular atrophy: a multicenter case series

**DOI:** 10.1186/s12883-025-04276-4

**Published:** 2025-07-07

**Authors:** Melissa D. Svoboda, Nancy Kuntz, Carmen Leon-Astudillo, Barry J. Byrne, Jena Krueger, Jennifer M. Kwon, Cory Sieburg, Diana Castro

**Affiliations:** 1 Division of Pediatric Neurology/Neurodevelopment, Department of Pediatrics, CHRISTUS Children’s/Baylor College of Medicine, San Antonio, TX USA; 2 Division of Neurology, Department of Pediatrics, Ann and Robert H. Lurie Children’s Hospital of Chicago, Northwestern University Feinberg School of Medicine, Chicago, IL USA; 3 Department of Pediatrics, Powell Gene Therapy Center, University of Florida College of Medicine, Gainesville, FL USA; 4 Division of Pediatric Neurology, Department of Pediatrics, Helen DeVos Children’s Hospital, Grand Rapids, MI USA; 5 Division of Pediatric Neurology, Department of Neurology, University of Wisconsin-Madison School of Medicine and Public Health, Madison, WI USA; 6 Neurology Rare Disease Center, Denton, TX USA

**Keywords:** Case series, Onasemnogene abeparvovec, Gene therapy, Risdiplam, Spinal muscular atrophy

## Abstract

**Background:**

Spinal muscular atrophy (SMA) is caused by deletions or mutations in the survival of motor neuron (SMN) 1 gene resulting in progressive motor function loss, and additional disease-related complications, including dysphagia and respiratory failure. With three US FDA–approved disease-modifying therapies (DMTs) available for SMA, patients, caregivers and healthcare providers have become increasingly interested in using a combination of DMTs to maximize clinical benefit. Current data on combination therapy are limited, and additional studies are needed.

**Case presentation:**

This multicenter, retrospective case series presents real-world outcomes in children with SMA who received onasemnogene abeparvovec (OA; ZOLGENSMA^®^), a single-dose gene therapy, and were subsequently treated with risdiplam (EVRYSDI^®^), a once-daily oral DMT. Adverse events as well as motor, respiratory and swallowing outcomes were evaluated before and after risdiplam initiation.

Twenty children were included, ten (50%) of whom were female. The majority had Type 1 SMA (*n* = 17; 85%) and two *SMN2* copies (*n* = 16; 80%). At baseline, eight (40%) children were clinically diagnosed with severe dysphagia, and ten (50%) required either noninvasive ventilation or invasive ventilation via tracheostomy. The mean time from OA administration to risdiplam initiation was 15.2 months, and the mean age at risdiplam initiation was 24.9 months. The most common reasons (*n* = 15; 75%) for starting risdiplam were either a plateau or inadequate improvement in disease symptoms. After risdiplam initiation, seven (35%) and six (30%) children had improvements in swallowing and respiratory function, respectively. Of the children whose motor function was assessed with the Children’s Hospital of Philadelphia Infant Test of Neuromuscular Disorders and/or the Hammersmith Functional Motor Scale – Expanded after risdiplam initiation, nearly all (*n* = 12/13; 92%) showed stability or improvement. No serious adverse events were observed post risdiplam initiation, and one child discontinued risdiplam due to a perceived lack of effectiveness.

**Conclusions:**

Many children included in this case series had improvements in motor, respiratory and/or bulbar function after adding risdiplam following OA. No new safety concerns were observed. The real-world evidence generated from this case series provides additional information on risdiplam’s risk–benefit profile after OA administration in children with SMA. Future studies with a larger cohort should be conducted.

**Graphical Abstract:**

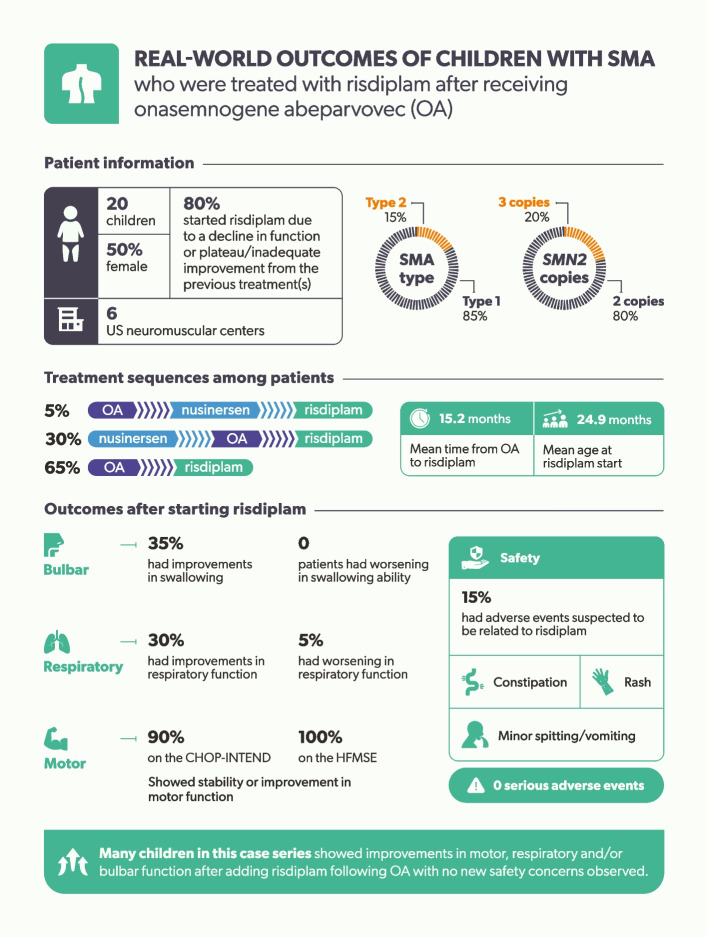

**Supplementary Information:**

The online version contains supplementary material available at 10.1186/s12883-025-04276-4.

## Background

Spinal muscular atrophy (SMA) is a genetic, progressive neuromuscular disease that leads to motor function loss and disease-related complications [1]. SMA is caused by biallelic deletions and/or mutations in the survival of motor neuron 1 (*SMN1*) gene, resulting in absent or reduced levels of functional SMN protein [1]. A second gene, survival of motor neuron 2 (*SMN2*), produces functional SMN protein but at a significantly lower level; *SMN2* cannot fully compensate for the loss of *SMN1* [1]. The disease course in patients with SMA is changing due to the wider implementation of newborn screening programs that provide earlier screening and diagnosis of SMA, and the availability of disease-modifying therapies (DMTs) [2, 3]. Three DMTs are approved by the US’ Food and Drug Administration for the treatment of SMA. In 2016, nusinersen (SPINRAZA^®^), an intrathecally administered *SMN2*-targeting antisense oligonucleotide therapy, was approved to treat children and adults with SMA [4]. In 2019, onasemnogene abeparvovec (OA; ZOLGENSMA^®^), a single-infusion, adeno-associated virus vector–based gene therapy that delivers a functional copy of *SMN1* to motor neurons, was approved to treat children aged < 2 years with SMA [5]. In 2020, risdiplam (EVRYSDI^®^), an orally administered *SMN2* pre-mRNA splicing modifier, was approved for the treatment of pediatric and adult individuals with SMA [6]. All three DMTs have shown clinical benefits in individuals with SMA; however, none offer a cure [7]. To further improve outcomes, physicians, along with individuals with SMA and their families, have sought combination therapy. One combination therapy of interest is risdiplam following OA administration. However, there are limited data on the safety and efficacy of risdiplam in patients previously treated with OA [8–11]. To date, JEWELFISH (NCT03032172) is the only clinical study investigating the safety and tolerability of risdiplam in patients who were previously treated with an SMA DMT [11]. Results from the primary analysis showed that risdiplam following OA (*n* = 14) was well tolerated, with no new safety concerns; however, the study was not designed to assess efficacy, and any captured efficacy data were exploratory [11]. The risdiplam expanded access program (NCT04256265) included nine individuals (5.8% of total enrollees) who received risdiplam following OA [8]. Although the expanded access program found that the safety profile of risdiplam was similar to that reported in the pivotal risdiplam clinical studies (FIREFISH, NCT02913482; SUNFISH, NCT02908685), the safety data were aggregated and not categorized by previous treatment; efficacy data were not collected. One retrospective case series reported the tolerability of risdiplam following OA in four individuals with Type 1 SMA [9]. The individuals tolerated the treatment combination and reported both subjective and objective improvements in motor function following risdiplam [9]. However, these results were from a small cohort of individuals at a single site with a short follow-up period following risdiplam initiation [9]. Lastly, a presentation at Cure SMA 2021 described the clinical presentation of four individuals with SMA who had received risdiplam following OA at a single site, and limited efficacy data were collected [10]. The data have not been published in a peer-reviewed manuscript.

This case series presents real-world outcomes in children with SMA who were treated with risdiplam after receiving OA.

## Methods

### Ethical approvals

Institutional review board approval was obtained or waived depending on the study site and local requirements. Informed consent was obtained from the parents or legal guardians of all patients unless the institution’s institutional review board waived this requirement. Data were anonymized/pseudonymized to ensure that data could not be linked to an individual.

### Patient population

This retrospective case series included 20 children with a genetically confirmed SMA diagnosis from six US neuromuscular centers. Patient demographic and clinical data were collected from the following sites: (1) Baylor College of Medicine/CHRISTUS Children’s Hospital (San Antonio, TX); (2) University of Florida College of Medicine (Gainesville, FL); (3) Helen DeVos Children’s Hospital (Grand Rapids, MI); (4) Ann and Robert H. Lurie Children’s Hospital of Chicago/Northwestern University Feinberg School of Medicine (Chicago, IL); (5) University of Wisconsin-Madison School of Medicine and Public Health (Madison, WI); and (6) Neurology Rare Disease Center (Denton, TX).

### Data collection

Patient demographics and clinical characteristics were collected from electronic health records (data cutoff: August 2023). Adverse events (AEs) and changes in dysphagia, respiratory function and motor function were described at baseline (before start of first SMA DMT) and after beginning each new SMA DMT. Dysphagia severity was determined based on a videofluoroscopic swallowing study, as available. When videofluoroscopic swallowing study was unavailable, dysphagia severity was classified as severe (g-tube dependent); moderate (mostly g-tube dependent with limited oral feeding); mild (mostly oral feeding with supplemental g-tube feeding or presence of any abnormalities with oral feeding); or no support (normal oral feeding).

Respiratory function was classified as invasive ventilation via tracheostomy, noninvasive ventilation (NIV) when awake and asleep, NIV only when asleep or no breathing assistance. Motor function was assessed using the Children’s Hospital of Philadelphia Infant Test of Neuromuscular Disorders (CHOP-INTEND) [12] and/or the Hammersmith Functional Motor Scale – Expanded (HFMSE) [13].

Descriptive statistics were used for all variables. Categorical data were presented as counts and percentages. Continuous or numerical data were presented as means and ranges. Patient or family reasons for starting a new SMA DMT were also collected and are categorized in Additional Table 1. A full list of clinical outcomes and adverse events at baseline, after OA and after risdiplam were tabularized in Additional Table 2 and Additional Table 3, respectively.

## Case presentation

### Patient information

**Fig. 1:**
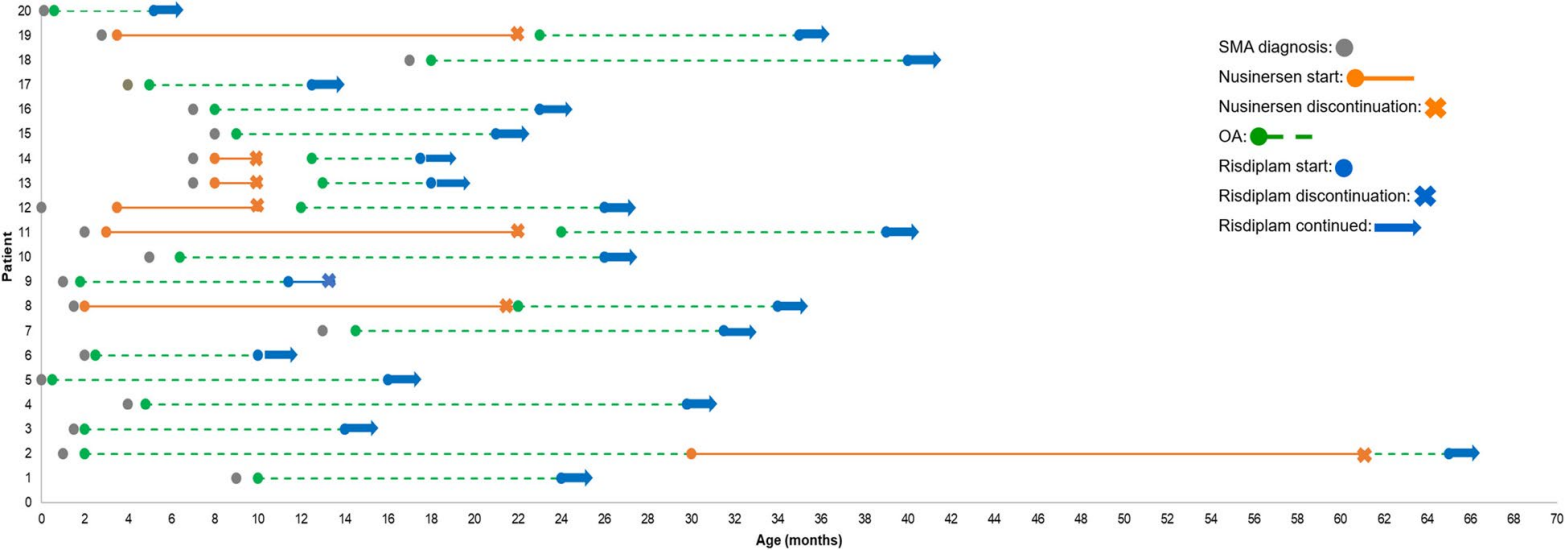
Age at SMA diagnosis and time to treatment. OA = onasemnogene abeparvovec; SMA = spinal muscular atrophy

Twenty children with SMA who received OA and subsequently risdiplam were included in this case series (Table 1). Most children had Type 1 SMA (85%) and two copies of *SMN2* (80%). The mean age at symptom onset was 2.5 months (range: 1 week–6.5 months), and the mean age at SMA diagnosis was 4.6 months (range: 0–17 months)​. Overall, the mean time from the administration of OA to risdiplam initiation was 15.2 months (range: 4.5–60 months), and the mean age at risdiplam initiation was 24.9 months (range: 5.2–65 months). The most common treatment sequence was OA then risdiplam (65%), followed by nusinersen, OA, then risdiplam (30%; Fig. 1). Of the six children who received nusinersen as their first DMT, most switched to OA due to their dislike of repeated lumbar punctures (67%) and desire for a single-dose treatment (67%). Common reasons for starting risdiplam after OA were inadequate improvement and plateau in improvement (75%). One child discontinued risdiplam due to perceived lack of effectiveness.

**Table 1:** Baseline characteristics

Baseline characteristics^a^	*N* = 20
**Sex, n (%)**	
Female	10 (50)
Male	10 (50)
**Mean age at symptom onset (range), months**	2.5 (0.2–6.5)
**Mean age at diagnosis (range), months**	4.6 (0–17)
***SMN2* copy, n (%)**	
2 copies	16 (80)
3 copies	4 (20)
**SMA type, n (%)**	
Type 1	17 (85)
Type 2	3 (15)
**Dysphagia, n (%)**^**b**^	
None	5 (25)
Mild	5 (25)
Moderate	2 (10)
Severe	8 (40)
**Baseline respiratory function, n (%)**	
No breathing assistance	10 (50)
NIV only when asleep	2 (10)
NIV awake and asleep	7 (35)
Tracheostomy/invasive ventilation	1 (5)
**Baseline motor function**	
Mean (range) baseline CHOP-INTEND score^c^	28.7 (20–45)

^a^Baseline characteristics were assessed before initiation of an SMA DMT. ^b^When available, a VFSS was used to determine the severity of dysphagia. In the absence of a VFSS, severity was defined as follows: severe dysphagia (g-tube dependent); moderate dysphagia (mostly g-tube dependent with limited oral feeding); mild dysphagia (mostly orally fed with supplemental g-tube feeding or presence of any abnormalities with oral feeding [e.g. increased secretions or increased length of feeding time]); and no support (normal oral feeding). ^c^Fifteen patients received a baseline CHOP-INTEND score

*CHOP-INTEND* Children’s Hospital of Philadelphia Infant Test of Neuromuscular Disorders, *DMT* disease-modifying therapy, *NIV* noninvasive ventilation, *SMA* spinal muscular atrophy, *SMN2* survival of motor neuron 2, *VFSS* videofluoroscopic swallowing study

### Dysphagia changes after risdiplam initiation

At baseline (before administration of an SMA DMT) and after receiving OA, eight children (40%) were diagnosed with severe dysphagia (Additional Fig. 1A; Additional Table 2). Of these children, three (38%) were able to take oral taste feeds after risdiplam. One child continued experiencing moderate dysphagia but was able to increase oral food intake after risdiplam. Three children who experienced mild dysphagia after receiving OA no longer had dysphagia symptoms after starting risdiplam, according to clinical assessment. Five children (25%) did not require any alternative ways of nutritional support at baseline, and of these five, none required support after risdiplam initiation.

### Respiratory changes after risdiplam initiation

Three children who previously required NIV after OA while awake and asleep only required NIV during sleep after receiving risdiplam (Additional Fig. 1B; Additional Table 2). One child who had required NIV during sleep after OA no longer required breathing assistance after starting risdiplam. After receiving risdiplam, two children had milder improvements in function and were able to decrease their NIV settings or increase their time off a ventilator. One child who did not require respiration assistance after OA began nighttime NIV after adding risdiplam, which was perceived to be due to SMA progression.

### Motor function after risdiplam initiation

**Fig. 2:**
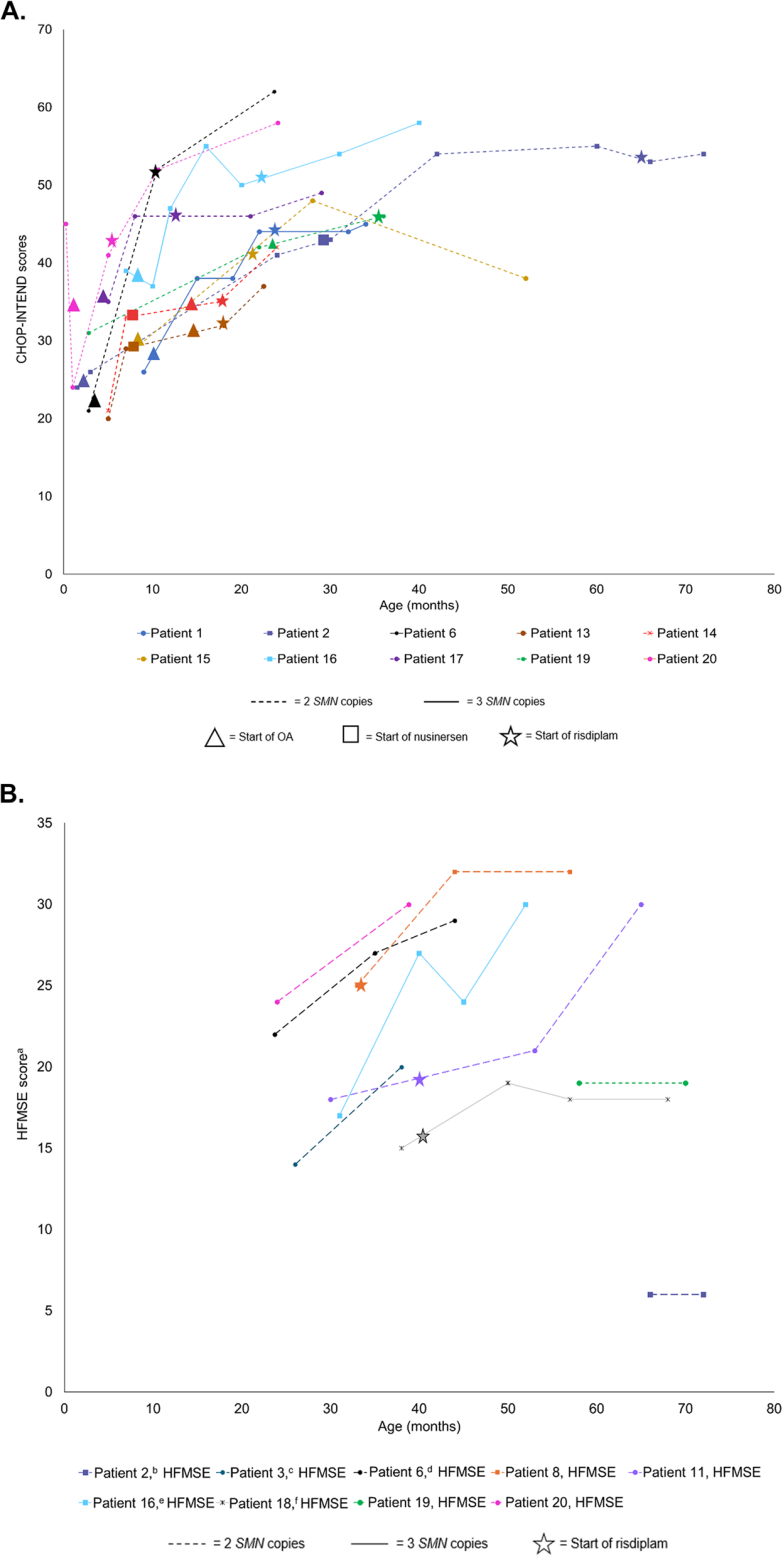
Motor function changes by (**A**) CHOP-INTEND and (**B**) HFMSE in patients after risdiplam initiation. Motor function changes in children who received a score after risdiplam initiation. ^a^Four children (patients 7, 10, 13 and 14) had only one HFMSE assessment performed. Patient 7 started risdiplam at 31.5 months and had an HFMSE score of 24 at 39 months; patient 10 started risdiplam at 26 months and had an HFMSE score of 2 at 40.8 months. Patient 13 started risdiplam at 18 months and had an HFMSE score of 10 at 29 months; patient 14 started risdiplam at 17.5 months and had an HFMSE score of 9 at 28.5 months. ^b^Patient 2 started risdiplam at 65 months. ^c^Patient 3 started risdiplam at 14 months. ^d^Patient 6 started risdiplam at 10 months. ^e^Patient 16 started risdiplam at 23 months. ^f^Patient 20 started risdiplam at 5.2 months. CHOP-INTEND = Children’s Hospital of Philadelphia Infant Test of Neuromuscular Disorders; HFMSE = Hammersmith Functional Motor Scale – Expanded; OA = onasemnogene abeparvovec; SMN = survival of motor neuron

Of the ten children whose motor function was assessed using CHOP-INTEND after risdiplam initiation, nine (90%) showed stability or improvement (Fig. 2A). Of the nine children who had more than one HFMSE assessment after risdiplam initiation, all showed stability or improvement (Fig. 2B).

### Adverse events after risdiplam initiation

Overall, four children (20%) experienced an AE after risdiplam (Additional Table 3). Of these four children, three (75%) experienced AEs suspected to be related to risdiplam treatment, including rash, constipation and minor spitting/vomiting. The event of rash and minor spitting/vomiting resolved with no changes to risdiplam treatment; however, the occurrence of constipation continued after risdiplam was discontinued due to inadequate functional improvement. One child experienced eczema, which was considered unrelated to risdiplam treatment and eventually resolved. One child discontinued risdiplam due to an AE (perceived lack of effectiveness). No serious AEs were observed after starting risdiplam.

### Highlight 1: patients 13 and 14

Patients 13 and 14 are twins who were diagnosed with Type 1 SMA at 7 months old and had two copies of *SMN2*. Both received nusinersen from ages 8 to 10 months but discontinued nusinersen due to their dislike of lumbar punctures and their desire for a single-dose treatment. Patients 13 and 14 received OA at 13 and 12.5 months, respectively; and began risdiplam at 18 and 17.5 months of age, respectively, due to a plateau in motor function. Dysphagia was severe at baseline and after OA, but both were tolerating oral taste feeds after risdiplam. At baseline and after OA, both children required respiratory support during wake and sleep; however, after risdiplam, both only required respiratory support during sleep. Patient 13’s CHOP-INTEND scores improved from 29 at baseline to 32 after OA and most recently to 37 after starting risdiplam. Patient 14’s CHOP-INTEND scores improved from 33 at baseline to 35 after OA and most recently to 42 after starting risdiplam. The perspective of the parent and their children’s experience with OA and risdiplam combination therapy can be found within the Additional file.

### Highlight 2: patient 19

Patient 19 was diagnosed with Type 1 SMA and two *SMN2* copies at age 2.8 months. Patient 19 received nusinersen from ages 3.5 to 22 months, received OA at 23 months and started risdiplam at 35 months due to poor oral secretion control. Dysphagia has remained severe from baseline through risdiplam treatment. Respiratory support has decreased from support needed while awake and sleep at baseline, to sleep only after OA, to no respiratory support required after risdiplam. In terms of motor function, CHOP-INTEND scores improved from 31 at baseline to 42 after nusinersen start. HFMSE score was not assessed in patient 19 before risdiplam initiation. After risdiplam start, they received an HFMSE score of 19. The perspective of the parent and their children’s experience with OA and risdiplam combination therapy can be found within the Additional file.

## Discussion

This case series aimed to increase understanding of the clinical benefit and safety profile of risdiplam after OA in patients with SMA. Plateaus in motor function improvement have been observed after treatment with SMA DMTs [14]. Therefore, combining DMTs for SMA treatment has been of interest, with the goal of maintaining or achieving maximal clinical benefit. In this case series, many children started risdiplam due to either a plateau in improvement or inadequate improvement. The effects of combination or sequential treatments of SMA DMTs are not yet well understood; however, evidence in a severe mouse model suggests combination therapy may increase the amounts of SMN protein, resulting in improved motor function and survival, even among symptomatic mice [15]. Nearly all the children in this case series were symptomatic prior to treatment, and most showed stability or improvements in motor function and made objective improvements in bulbar function and/or respiratory function after initiating risdiplam. However, there was no clear trend in outcomes based on SMA type, *SMN2* copy number or time to treatment. Our case series showed that children treated with risdiplam after OA experienced improvements in motor function and that treatment appeared to be well tolerated, with no significant safety concerns. This is similar to findings of other case reports and clinical studies that showed that children treated with OA and subsequently risdiplam experienced overall improvement, with no new safety concerns [8–11].

### Limitations

This was a small case series that focused on six neuromuscular centers; thus, these results may not be generalizable. Future studies should expand recruitment and focus outside of large neuromuscular centers in the United States to further evaluate risdiplam safety and effectiveness following OA administration. Although overall improvement was observed, it is difficult to differentiate whether OA, risdiplam or both are the source of continued improvement. Notably, swallowing and respiratory function changes were assessed primarily through clinical evaluations. Objective instrumental evaluations of swallowing function, as well as polysomnographic studies, were not routinely performed at all sites. Additionally, differing assessment timings and the motor tests administered may contribute to data variability.

## Conclusions

Most children in this case series started risdiplam approximately 15.2 months after OA, which was most commonly due to inadequate improvement or a plateau in improvement. Many had objective improvements in motor skills, bulbar function and/or respiratory function after adding risdiplam. After starting risdiplam, some children demonstrated improvement that was beyond what would be expected if no additional treatments were started. Risdiplam after OA appears to be well tolerated, with no significant AEs reported. Overall, these data show the promising benefit of combination therapy for the treatment of SMA. Studies with larger cohorts of patients are underway to provide more insight into the safety and effectiveness of risdiplam following OA [16, 17].

## Supplementary Information

Supplementary Material 1.

## Data Availability

Data generated and/or analyzed in this study are not available due to privacy and ethical restrictions and cannot be shared publicly or transmitted to a third party.

## Abbreviations

AE: Adverse event
CHOP-INTEND: Children’s Hospital of Philadelphia Infant Test of Neuromuscular Disorders
DMT: Disease-modifying therapy
HFMSE: Hammersmith Functional Motor Scale – Expanded
NIV: Noninvasive ventilation
OA: Onasemnogene abeparvovec
SMA: Spinal muscular atrophy
SMN1: Survival of motor neuron 1
SMN2: Survival of motor neuron 2
