# Supplementary material for: Risdiplam treatment following onasemnogene abeparvovec in individuals with spinal muscular atrophy: a multicenter case series
Source: BMC Neurol. 2025 Jul 7;25:283. doi: 10.1186/s12883-025-04276-4 (PMC12232808; doi:10.1186/s12883-025-04276-4)
Supplement: Supplementary file 1 — Supplementary Material 1. [file 12883_2025_4276_MOESM1_ESM.docx]

**Supplementary Materials**

**Risdiplam treatment following onasemnogene abeparvovec in individuals with spinal muscular atrophy: a multicenter case series**

Melissa D. Svoboda^1*^, Nancy Kuntz^2^, Carmen Leon-Astudillo^3^, Barry J. Byrne^3^, Jena Krueger^4^,
Jennifer M. Kwon^5^, Cory Sieburg^5^, Diana Castro^6^

^1^Division of Pediatric Neurology/Neurodevelopment, Department of Pediatrics, CHRISTUS Children's/Baylor College of Medicine, San Antonio, Texas

^2^Division of Neurology, Department of Pediatrics, Ann and Robert H. Lurie Children's Hospital of Chicago, Northwestern University Feinberg School of Medicine, Chicago, Illinois

^3^Department of Pediatrics, University of Florida College of Medicine, Gainesville, Florida

^4^Division of Pediatric Neurology, Department of Pediatrics, Helen DeVos Children's Hospital, Grand Rapids, Michigan

^5^Division of Pediatric Neurology, Department of Neurology, University of Wisconsin-Madison School of Medicine and Public Health, Madison, Wisconsin

^6^Neurology Rare Disease Center, Denton, Texas

*Corresponding author: melissa.svoboda@bcm.edu

**Parent perspectives**

**Perspective for patients 13 and 14**

The parents provided the following perspective when asked about their experience and their children’s experience with OA and risdiplam combination therapy:

*“The change risdiplam has brought to my twin boys’ lives is beyond words. The difference it made is huge and we see them getting better and stronger each day. Risdiplam has significantly improved their quality of life by enhancing their mobility, boosting their independence, and fostering a greater sense of confidence allowing them to engage more with their peers and family.*

*Before beginning treatment, they struggled significantly with respiratory weakness, mobility, and independence. Since starting risdiplam, we have observed remarkable improvements in their physical capabilities; their enhanced mobility has not only allowed them to navigate spaces with greater ease but also to partake more actively in various activities that were previously beyond their reach.”*

**Perspective for patient 19**

The parents provided the following perspective when asked about their experience and their child’s experience with OA and risdiplam combination therapy:

*“Our child, diagnosed with Type 1 SMA at three months of age, was given an initial prognosis of six months to a year. As we are gearing up to celebrate our child’s seventh birthday, we know that this would not have been possible without the dual treatments for their SMA. Not only are they beating the odds but also growing and thriving despite having an SMA diagnosis.*

*Our family adamantly believes that dual therapies for our child's SMA are the reason for growth in pulmonary function. Our child, Type 1 SMA with lung collapse at age 3 months and sent home from the pediatric intensive care unit at 7 months with a tracheostomy and ventilator, no longer requires a ventilator, even for procedures under anesthesia. These dual treatments have provided our child the strength to breathe independently. Because of these treatments, our medical team is now taking steps towards decannulation.*

*While ZOLGENSMA provided our child with an infusion of new strength, the daily risdiplam has taken strength to the next level. Our child’s strength only continues to grow to this day, and we are certain it is because they had the benefit of both treatments”.*

**Additional Figure 1. (A) Dysphagia changes^a^ and (B) respiratory changes^b^**

**
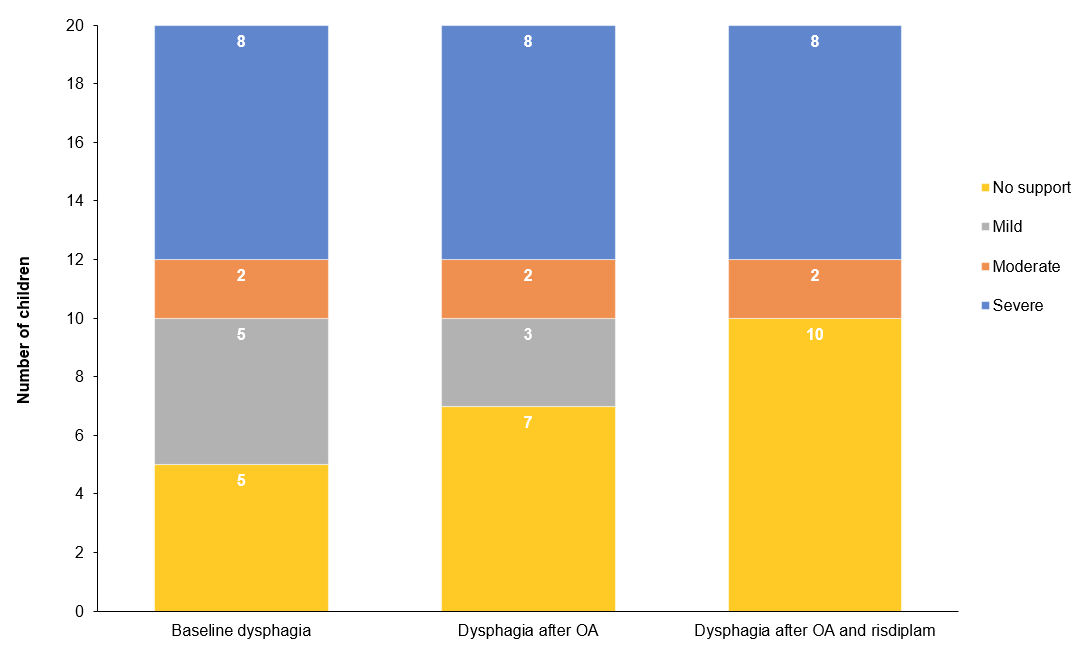
**

**B.

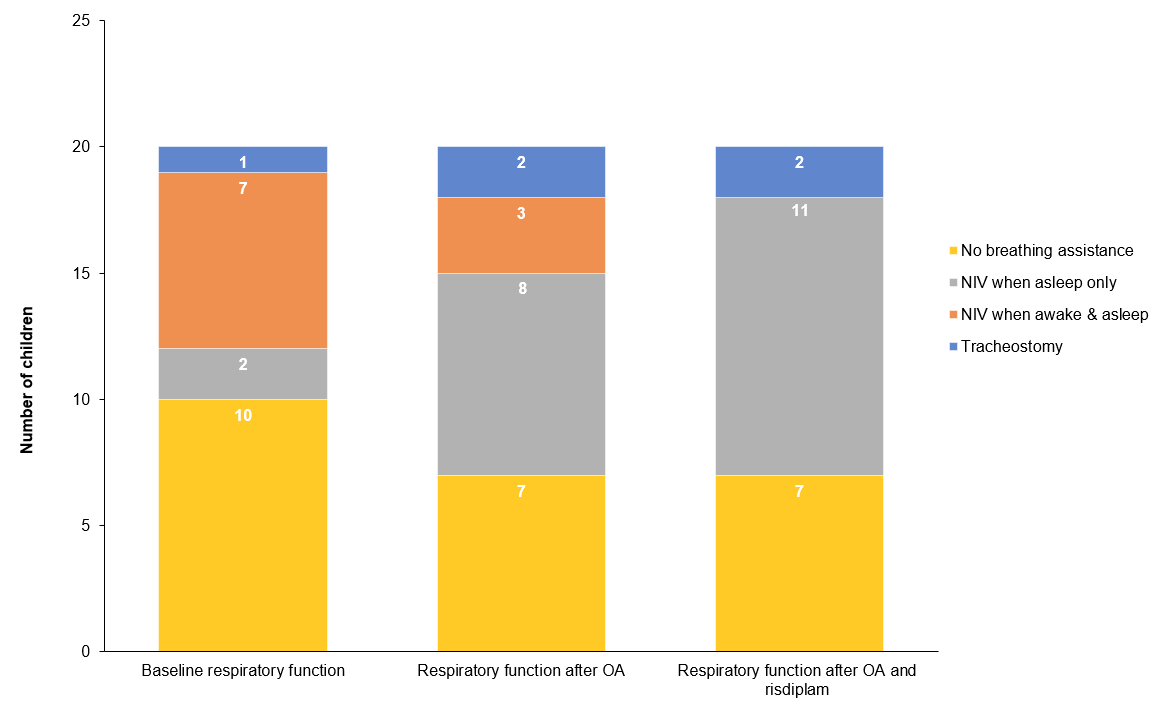
**

^a^When available, a VFSS was used to determine the severity of dysphagia. In the absence of a VFSS, severity was defined as follows: severe dysphagia (g-tube dependent); moderate dysphagia (mostly g-tube dependent with limited oral feeding); mild dysphagia (mostly orally fed with supplemental g-tube feeding or presence of any abnormalities with oral feeding [e.g. increased secretions or increased length of feeding time]); and no support (normal oral feeding). ^b^Some reasons for NIV treatment included difficulties with secretions and respiratory weakness. NIV = noninvasive ventilation; OA = onasemnogene abeparvovec; VFSS = videofluoroscopic swallowing study.

**Additional Table 1. Reasons for starting treatment**

| **​Patient** | **Treatment with nusinersen** | **Reasons for starting OA** | **Reason for starting risdiplam​** | **Risdiplam initiation category** |
| --- | --- | --- | --- | --- |
| **1**​ | - | The child was brought to the emergency department at age 9 months with symptoms of SMA | The child continued having very low muscle tone and difficulties with secretion management​ | Inadequate improvement​^a^ |
| **2**​^b^ | Nusinersen was started 3 years after OA | Experimental gene delivery therapy was administered; the child was treated with nusinersen following gene therapy | The child had a limited response with nusinersen and continued having very low muscle tone and inability to walk ​ | Inadequate improvement​ |
| **3**​ | - | The child was aged <2 years and symptomatic since age 1 month ​ | The child had severe hypotonia, severe scoliosis and limited motor function such as rolling over​ | Inadequate improvement​ |
| **4**​ | - | OA could be administered as a single dose, and promising data from a trial were present | The goal was to improve function and decrease respiratory support | Inadequate improvement |
| **5**​^c^ | - | The family wanted a single-dose medication​ | Family request​ | Family request​ |
| **6**​ | - | After diagnosis of Type 1 SMA, the parents requested gene therapy​ | The child seemed to be making slow motor gains​ | Inadequate improvement​​ |
| **7**​ | - | After diagnosis of Type 2 SMA, the parents wanted gene therapy​ | The mother requested risdiplam when the child was aged 3 years ​ | Family request​ |
| **8**​ | Nusinersen was received at 8 weeks | There was a desire to stop maintenance nusinersen (LPs every 4 months)​ | Did not feel that gene therapy was effective given the development of TMA​ ​ | Inadequate improvement​ |
| **9**​ | - | The goal was to treat SMA, per the parents' request​ | Did not feel that gene therapy was that effective​ | Inadequate improvement​ |
| **10**​ | - | The diagnosis of SMA occurred during hospitalization, and a single-dose treatment was desired​ | The treatment was recently approved​ | Treatment approved​ |
| **11**​ | Nusinersen was received at 3 months | OA was approved; a single-dose treatment was desired, and the child disliked LPs ​ | A plateau in motor skill progression​ was observed | Plateau in improvement​^d^ |
| **12**​ | Nusinersen was received at 3.5 months | A single-dose treatment was desired, and promising data from a trial were present ​ | A plateau in motor skill progression​ was observed | Plateau in improvement​ |
| **13**​ | Nusinersen was received at 8 months | A single-dose treatment was desired, and the child disliked LPs​ | A plateau in motor skill progression occurred, and the mother read about combination therapy and improvements​ online | Plateau in improvement​ |
| **14**​ | Nusinersen was received at 8 months | A single-dose treatment was desired, and the child disliked LPs ​ | A plateau in motor skill progression was observed, and the mother read about combination therapy and improvements​ online | Plateau in improvement​ |
| **15** | - | The child experienced respiratory failure and wanted a treatment for SMA | The family wanted to pursue  combination therapy to  improve motor milestones | Inadequate improvement​ |
| **16** | - | The child began a treatment for SMA | The child experienced decreased motor milestones | Decline in function |
| **17** | - | The child began a treatment for SMA | The family wanted to pursue dual therapy | Family request |
| **18** | - | Disease progression occurred | Adding SMN protein enhancing medication might improve function and reduce hospitalizations | Inadequate improvement |
| **19** | Nusinersen was received at 3.5 months | The child had fragile bulbar and respiratory status | The child had major problems with oral secretions that required constant suctioning | Inadequate improvement​ |
| **20** | - | OA was given prophylactically | The parents noted slow gains in function and poor weight gain | Inadequate improvement |

^a^A patient was classified as experiencing inadequate improvement if they experienced some improvement but not as much as they had hoped.

^b^Patient 2 received nusinersen for 2 years. ^c^Patient 5 was presymptomatic before treatment with OA; all other patients were symptomatic at the time of gene therapy. ^d^A patient was classified as experiencing a plateau in improvement if clinical outcomes did not improve over time (i.e. if motor skills did not progress). LP = lumbar puncture; OA = onasemnogene abeparvovec; SMA = spinal muscular atrophy; SMN = survival of motor neuron; TMA = thrombotic microangiopathy.

**Additional Table 2. Clinical outcomes at baseline, after OA and after risdiplam**

| **Patient​** | **Dysphagia​^a^** | | | **Respiratory support** | | | **Motor ​** | | |
| --- | --- | --- | --- | --- | --- | --- | --- | --- | --- |
|  | **Baseline** | **After OA** | **After risdiplam start** | **Baseline** | **After OA** | **After  risdiplam start** | **Baseline** | **After OA** | **After risdiplam start** |
| 1​ | Mild | Severe | Severe | Sleep only | Sleep only | Sleep only;  NIV settings decreased | CHOP-INTEND score of 26 at age 9 months | CHOP-INTEND score of 38 at age 15 months; CHOP-INTEND score of 38 at age 19 months; and CHOP-INTEND score of 44 at age 22 months | CHOP-INTEND score of 44 at age 32 months and CHOP-INTEND score of 45 at age 34 months |
| 2​ | Severe | No  support | No  support | No breathing assistance | No breathing assistance | No breathing assistance | CHOP-INTEND score of 24 at age 1.5 months | CHOP-INTEND score of 26 at age 3 months; CHOP-INTEND score of 41 at 24 months; CHOP-INTEND score of 43 at 30 months; CHOP-INTEND score of 54, , RULM score of 11 at 42 months; RULM score of 13 at 48 months; CHOP-INTEND score of 55, RULM score of 15 at 60 months; CHOP-INTEND score of 53, RULM score of 16; HFMSE score of 6 and RHS score of 6 at 66 months | CHOP-INTEND score of 54, HFMSE score of 6 and RHS score of 6 at 72 months |
| 3​ | No  support | No  support | No  support | No  breathing assistance | No  breathing assistance | No breathing assistance | CHOP-INTEND score of 33 | CHOP-INTEND score of 48 at age 14 months | HFSME score of 14 at age 26 months and 20 at age 38 months; RHS score of 10 at age 26 months and 13 at age 38 months |
| 4 | Severe | Severe | Severe but now tolerating taste feeds | No  breathing assistance | Tracheostomy/ventilator | Tracheostomy/ventilator; now tolerates time off ventilator | Tongue fasciculations; severe hypotonia throughout; head lag; slip-through with vertical suspension; draping with horizontal suspension; ability to lift left elbow off table but inability to reach above shoulder level on either side; inability to bear weight; when prone, collapse without lifting head; absent tendon reflexes besides biceps; CHOP-INTEND score of 30 (left) and 24 (right) at age 4.4 months; and CHOP-INTEND score of 30 (left) and 24 (right) at age 4.5 months | CHOP-INTEND score of 33 (left) and 28 (right) at age 5.1 months;  CHOP-INTEND score of 41 (left) and 40 (right) at age 7.6 months; CHOP-INTEND score of 40 (left) and 39 (right) at age 16.7 months;  NeuroGRO score of 20 at age 16.7 months; NeuroGRO score of 17 at age 18.1 months; NeuroGRO score of 17 at age 17.9 months; and NeuroGRO score of 15 at age 23.5 months | NeuroGRO score of 20 at age 32.3 months; NeuroGRO score of 21 at age 35.4 months; NeuroGRO score of 20 at age 39.6 months; NeuroGRO score of 24 at age 44.2 months; and NeuroGRO score of 24 at age 48.0 months |
| 5​ | No  support | No  support | No  support | No  breathing assistance | No  breathing assistance | No  breathing assistance | No formal scales used; examination at 3 days of life revealed largely typical motor function with antigravity movement of arms and legs; more hypotonic axial tone; head lag but not particularly outside the normal range; and present deep tendon reflexes | Feeding well; antigravity movement of arms and legs; better head control; no formal scales used; CHOP-INTEND score of 57 at age 3 months; and CHOP-INTEND score of 56 at age 11.8 months | PDMS-2 score in the 5th percentile, with GMQ score of 76 at age 25 months |
| 6​ | No  support | Mild | No  support | No  breathing assistance | No  breathing assistance | Sleep only | Weakness with antigravity movement of forearms; leg weakness without antigravity movement; and head lag | CHOP-INTEND score of 21 at age 2.8 months and CHOP-INTEND score of 52 at age 10.5 months | CHOP-INTEND score of 62 and HFMSE score of 22 at age 23.7 months; HFMSE score of 27 at age 35 months; and HFMSE score of 29 at age 44 months |
| 7​ | No  support | No  support | No  support | No  breathing assistance | No  breathing assistance | No breathing assistance | Ability to sit unsupported for 5 seconds and inability to crawl | No formal assessment | Knee contractures (-15 to 20 degrees in extension); use of self-propelling, lightweight, manual wheelchair; and HFMSE score of 24 at age 39 months |
| 8​ | Mild | No  support | No  support | Awake and asleep | Sleep only | Sleep only | Ability to lift arms; inability to lift legs; and absent reflexes at age 1.3 months | Ability to sit unsupported and raise arms to face and over the head; HFSME score of 25 at age 33 months; and CHOP-INTEND score of 56 at age 33 months | HFMSE score of 32 at age 44 months and HFMSE score of 32 at age 57 months |
| 9​ | Mild | Mild | No  support | Awake and asleep | Sleep only | Sleep only | Severe hypotonia with limited arm movements; no antigravity movements of limbs; CHOP-INTEND score of 20 at age 2 months | CHOP-INTEND score of 22 at age 11 months | Ability to sit unsupported, roll and use gait trainer to take steps at age 42 months |
| 10​ | Moderate | Severe | Severe | Tracheostomy/ventilator | Tracheostomy/ventilator | Tracheostomy/ventilator | Tongue fasciculations; diffuse hypotonia; ability to move wrists and ankles; inability to move shoulders, hips or knees; absent reflexes throughout; and CHOP-INTEND score of 18 (left) and 22 (right) at age 6.3 months | Improvement in the postachilles tenotomy on the right; CHOP-INTEND score of 23 (left) and 20 (right) at age 8 months, 16 (left) and 16 (right) at age 8.9 months, 35 (left) and 30 (right) at age 16.4 months and 34 (left) and 34 (right) at age 25.5 months; and  NeuroGRO score of 15 at age 16.4 months and 18 at age 25.5 months | Axial hypotonia; head lag; slip-through; ability to lift arms overhead when supine and to the shoulder when support seated (improved from prior); ability to maintain feet flat on mat with both knees upright; NeuroGRO score of:20 at age 34.6 months of age;  significant scoliosis; plagiocephaly; subtle tongue fasciculations; ability to maintain feet flat on mat with both knees upright; inability to bear weight; good head control; no reflexes; inability to ambulate; and  RHS score of 1, HMFSE score of 2 and NeuroGRO score of 23 at age 40.8 months |
| 11​ | Severe | Moderate | Moderate | No breathing assistance | Awake and asleep | Sleep only | Severe hypotonia; weakness with no head control; ability of arms to move against gravity at the elbows; ability to only kick out to side; HINE-2 score of 1 at age 2 months; HINE-2 score of 5 at age 8 months; and CHOP-INTEND score of 44 at age 20 months | HFMSE score of 18 at age 30 months | HFMSE score of 21 at age 54 months and HFMSE score of 30 at age 65 months |
| 12​ | Mild | No  support | No  support: The mother communicated that the child is doing “awesome”; dysphagia has improved. | No breathing assistance | No breathing assistance | No breathing assistance: the child has had no breathing issues or hospitalizations this year | CHOP-INTEND score of 21 at age 3 months | CHOP-INTEND score of 50 at age 25 months | The child can scoot on her bottom in the bathtub |
| 13​ | Severe | Severe | Severe but now tolerating taste feeds | Awake and asleep | Awake and asleep | Sleep only | CHOP-INTEND score of 20 at age 5 months and CHOP-INTEND score of 29 at age 7 months | The child's mother is disappointed with the slow improvement; CHOP-INTEND score of 32 at age 18 months | CHOP-INTEND score of 37 at age 23 months and HFMSE score of 10 at age 29 months |
| 14​ | Severe | Severe | Severe but now tolerating taste feeds | Awake and asleep | Awake and asleep | Sleep only | CHOP-INTEND score of 21 at age 5 months and CHOP-INTEND score of 33 at age 7 months | The PT and OT noticed significant plateau for 2 months straight without further improvements; CHOP-INTEND score of 35 at age 17.5 months | Ability to sit independently for 15 minutes; CHOP-INTEND score of 42 at age 21.5 months; and HFMSE score of 9 at age 28.5 months |
| 15 | Severe | Severe | Severe | Sleep only | Sleep only | Sleep only | Diffuse hypotonia with significant head lag; ability to move arms; ability to bring hands to mouth; ability to only move legs in plane of bed and not against gravity; and CHOP-INTEND score of 29 and HINE-2 score of 2 at age 8 months | Ability to sit and HINE-2 score of 5 at age 15 months | Ability to bring hips to abdomen, extend knees and give small push to the physician; ability to bring arms above head; CHOP-INTEND score of 48 and HINE-2 score of 6 at age 28 months; and CHOP-INTEND score of 38 at age 52 months |
| 16 | Severe | Moderate | Moderate; increased oral food intake | Awake and asleep | Sleep only | Sleep only | Inability to sit up; inability to bring head up from supine position or reach above head; inability to flex at hips with the lower extremities; and CHOP-INTEND score of 39 at age 7 months | Observed initial improvement, then decline occurred; CHOP-INTEND score of 37 at age 10 months, 47 at age 12 months, 55 at age 16 months and 50 at age 20 months | Improved motor skills; louder voice; more active; ability to sit unsupported; CHOP-INTEND score of 54 and HMFSE score of 17 at age 31 months;  CHOP-INTEND score of 58 and HFMSE score of 27 at age 40 months;  HFMSE score of 24 at age 45 months; and HFMSE score of 30 at age 52 months |
| 17 | Moderate | Severe | Severe | Awake and asleep | Sleep only | Sleep only | CHOP-INTEND score of 35 at age 5 months | Improved motor skills and head control; ability to sit for short periods of time at age 8 months; and CHOP-INTEND score of 46 at age 8 months | Improved motor functions and louder voice; CHOP-INTEND score of 46 at age 21 months; and CHOP-INTEND score of 49 at age 29 months |
| 18 | No support | No  support | No support | No breathing assistance | Sleep only | Sleep only | Hypotonia and severe motor delay; inability to lift head during tummy time or roll over; and inability to push into seated position independently | Ability to flex at the hips better and abduct at the shoulders better; ability to strengthen trunk; HFMS score of 7 at age 26 months; HFMS score of 10 at age 33 months; and HFMS score of 13 and HFMSE score of 2/26 (sum: 15/66) at age 39 months | Ability to sit without assistance; ability to reach up to approximately 100° with the arms; improved stability of head control but inability to stand;  HFMSE score of 19 at age 50 months, 18 at age 57 months, 18 at age 68 months and 2 (from 0-6 on upper extremity scale) at age 74 months |
| 19 | Severe | Severe | Severe | Awake and asleep | Sleep only | No breathing assistance | CHOP-INTEND score of 31 at age 2.8 months; CHOP-INTEND score of 42; WHO motor milestones score of 1; HINE score of 9 at age 22 months | Voice stronger at age 25 months; CHOP-INTEND score of 46; HINE score of 9 at age 36 months | HFMSE score of 19 at age 58 months; WHO motor milestones score of 1; HINE score of 13; Bayley-12 fine motor score of 1; Bayley-12 gross motor score of 13; and RULM score of 7 (left) and 8 (right) at age 58 months; ability to bear weight and stand briefly when placed on the ground at age 64 months; HFMSE score of 19 at age 70 months; WHO motor milestones score of 1; HINE score of 16; and RULM score of 14 (L) and 17 (R) |
| 20 | Mild | Mild | No support | No breathing assistance | No breathing assistance | No breathing assistance | At age 0.23 months:  Bayley: fine motor raw 3, fine motor scaled 0, gross motor raw 4, gross motor scaled 0, composite 46 and percentile 0.1%; CHOP-INTEND score of 45;  WHO motor milestones included inability to sit without support; no hands-and-knees crawling; inability to stand or walk with assistance; and inability to stand or walk alone; HINE-2: voluntary grasp (uses whole hand); ability to kick (in supine): vertical; wobbly head control; no rolling, sitting, standing, walking or crawling (does not lift head) | At age 0.82 months, the parents report that the child has poor head control and less movement of the legs and arms.  At age 1 month: Bayley: fine motor raw 3, fine motor scaled 0, gross motor raw 4, gross motor scaled 0, composite 46 and percentile 0.1%; CHOP-INTEND score of 24; WHO motor milestones: inability to sit without support; no hands-and-knees crawling; inability to stand or walk with assistance; and inability to stand or walk alone HINE-2: voluntary grasp (uses whole hand); ability to kick (in supine): vertical; wobbly head control; no rolling, sitting, standing or walking; inability to lift head  At age 5 months: Bayley: fine motor raw 7, fine motor scaled 4, gross motor raw, gross motor scaled 5, composite 67 and percentile 1; CHOP-INTEND score of 41; WHO motor milestones: inability to sit without support; no hands-and-knees crawling; inability to stand or walk with assistance; and inability to stand or walk alone  HINE-2: voluntary grasp (uses whole hand); ability to kick (in supine): horizontal; inability to lift legs ; upright head control; no rolling, sitting, standing or walking; and ability to lift head while crawling | At age 10.6 months: Bayley: fine motor raw 23, fine motor scaled 7, gross motor 17, gross motor scaled, composite 64 and total scaled 8; CHOP-INTEND score of 52  At age 24.1 months:  CHOP-INTEND score of 58 and HFMSE score of 24  At age 38.8 months:  HFMSE score of 30 and ability to stand and walk independently |

CHOP-INTEND, Children’s Hospital of Philadelphia Infant Test of Neuromuscular Disorders; GMQ, gross motor quotient; HFMS, Hammersmith Functional Motor Scale; HFMSE, Hammersmith Functional Motor Scale – Expanded; HINE, Hammersmith Infant Neurological Examination; NeuroGRO, Neuromuscular Gross Motor Outcome; NIV; noninvasive ventilation; OA, onasemnogene abeparvovec; OT, occupational therapist ; PDMS-2, Peabody Developmental Motor Scales Second Edition; PT, physical therapist ; RHS, Revised Hammersmith Scale; RULM, Revised Upper Limb Module; VFSS, videofluoroscopic swallowing study; WHO, World Health Organization. ^a^When available, a VFSS was used to determine the severity of dysphagia. In the absence of a VFSS, severity was defined as follows: severe dysphagia (g-tube dependent); moderate dysphagia (mostly g-tube dependent with limited oral feeding); mild dysphagia (mostly orally fed with supplemental g-tube feeding or presence of any abnormalities with oral feeding [e.g. increased secretions or increased length of feeding time]); and no support (normal oral feeding).

**Additional Table 3. Baseline AEs and AEs after treatment**

| **Patient** | **Baseline AEs** | **AEs after gene therapy** | **AEs after risdiplam** |
| --- | --- | --- | --- |
| 1 | None | Vomiting and elevation of transaminases | None |
| 2 | None | Vomiting and elevation of transaminases | None |
| 3 | None | Elevation of transaminases | None |
| 4 | None | None | None |
| 5 | None | None | None |
| 6 | None | Disease progression leading to hospitalization | None |
| 7 | None | Elevated transaminase and GGT levels 4 weeks after treatment; continued full dose of prednisolone (1 mg/kg) for additional 4 weeks with slow taper off steroids | None |
| 8 | None | Steroid-induced bradycardia; thrombotic angiopathy^a^ with nephrotic syndrome and hypertension | Developed a rash for less than a week, which was likely risdiplam related; resolved with no topical agent or medication changes |
| 9 | None | None | Constipation after 2 months of treatment, which was suspected to be risdiplam related; since discontinuing risdiplam due to inadequate functional improvement, constipation has continued |
| 10 | Respiratory failure/pneumonia and rhinovirus infection | None | None |
| 11 | Severe failure to thrive | None | None |
| 12 | None | Prolonged elevation of transaminases | Eczema, which was classified as unrelated to treatment and resolved |
| 13 | None | None | None |
| 14 | None | Slight elevation in troponin levels, which resolved | None |
| 15 | None | None | None |
| 16 | None | None | None |
| 17 | Pneumonia | Slow weight gain | None |
| 18 | None | None | None |
| 19 | Bad reflux and pneumonia | None | Minor spitting and vomiting for 3–4 days,  which were suspected to be risdiplam related and resolved with continued treatment |
| 20 | None | None | None |

^a^A known complication of gene therapy [1]. AE = adverse event; GGT = gamma-glutamyl transferase.

1. Chand DH, Zaidman C, Arya K, Millner R, Farrar MA, Mackie FE, et al. Thrombotic microangiopathy following onasemnogene abeparvovec for spinal muscular atrophy: a case series. J Pediatr. 2021;231:265-8.
